# EC-PGMGR: Ensemble Clustering Based on Probability Graphical Model With Graph Regularization for Single-Cell RNA-seq Data

**DOI:** 10.3389/fgene.2020.572242

**Published:** 2020-11-04

**Authors:** Yuan Zhu, De-Xin Zhang, Xiao-Fei Zhang, Ming Yi, Le Ou-Yang, Mengyun Wu

**Affiliations:** ^1^School of Automation, China University of Geosciences, Wuhan, China; ^2^Hubei Key Laboratory of Advanced Control and Intelligent Automation for Complex Systems, Wuhan, China; ^3^Department of Statistics, School of Mathematics and Statistics, Central China Normal University, Wuhan, China; ^4^School of Mathematics and Physics, China University of Geosciences, Wuhan, China; ^5^Guangdong Key Laboratory of Intelligent Information Processing and Shenzhen Key Laboratory of Media Security, Shenzhen University, Shenzhen, China; ^6^School of Statistics and Management, Shanghai University of Finance and Economics, Shanghai, China

**Keywords:** single-cell, ensemble clustering, probability graphical model, graph regularization, non-negative matrix factorization

## Abstract

Advances in technology have made it convenient to obtain a large amount of single cell RNA sequencing (scRNA-seq) data. Since that clustering is a very important step in identifying or defining cellular phenotypes, many clustering approaches have been developed recently for these applications. The general methods can be roughly divided into normal clustering methods and integrated (ensemble) clustering methods which combine more than two normal clustering methods aiming to get much more informative performance. In order to make a contrast with the integrated clustering algorithm, the normal clustering method is often called individual or base clustering method. Note that the results of many individual clustering methods are often developed to capture one aspect of the data, and the results depend on the initial parameter settings, such as cluster number, distance metric and so on. Compared with individual clustering, although integrative clustering method may get much more accurate performance, the results depend on the base clustering results and integrated systems are often not self-regulation. Therefore, how to design a robust unsupervised clustering method is still a challenge. In order to tackle above limitations, we propose a novel Ensemble Clustering algorithm based on Probability Graphical Model with Graph Regularization, which is called EC-PGMGR for short. On one hand, we use parameter controlling in Probability Graphical Model (PGM) to automatically determine the cluster number without prior knowledge. On the other hand, we add a regularization term to reduce the effect deriving from some weak base clustering results. Particularly, the integrative results collected from base clustering methods can be assembled in the form of combination with self-regulation weights through a pre-learning process, which can efficiently enhance the effect of active clustering methods while weaken the effect of inactive clustering methods. Experiments are carried out on 7 data sets generated by different platforms with the number of single cells from 822 to 5,132. Results show that EC-PGMGR performs better than 4 alternative individual clustering methods and 2 ensemble methods in terms of accuracy including Adjusted Rand Index (ARI) and Normalized Mutual Information (NMI), robustness, effectiveness and so on. EC-PGMGR provides an effective way to integrate different clustering results for more accurate and reliable results in further biological analysis as well. It may provide some new insights to the other applications of clustering.

## 1. Introduction

Cells are considered as the most basic functional units of an organism (Rosvall and Bergstrom, 2017). The identification of cell types has a great impact on the discovery of novel cells and the study of cell function (Trapnell et al., 2014a; Reid et al., 2018). In fact, the expression levels of most genes vary widely among different cell types in different cells. Bulk-RNA sequencing (RNA-seq) is a technology that averages the expression levels across many cells from different cell types, which may conceal some meaningful expression information (Trapnell et al., 2014b; Yang et al., 2018). Comparatively, single-cell RNA sequencing (scRNA-seq) characterizes the heterogeneity of cells, and is able to identify novel cell types, predict cell fate, and classify tumor subpopulation from a finer resolution perspective (Jia et al., 2017; Treutlein et al., 2014). Clustering is a very important step in the above applications. With the clustering results in hand, it is convenient and meaningful to analyze different expressions in down stream.

Thus, a lot of clustering techniques including individual clustering and ensemble clustering are proposed for cell clustering with scRNA-seq. Examples include t-Distributed Stochastic Neighbour Embedding algorithm (t-SNE) by *k*-means clustering (Dominic et al., 2015), Seurat (Satija et al., 2015), SIMLR (Wang et al., 2017), SC3 (Kiselev et al., 2017), and SCANPY (Wolf et al., 2018). The results of these clustering methods vary due to different settings of distance metric and initial input of probably cluster number and so on. For example, the result of SC3 depends highly on the input setting of cluster number. However, in practical scientific research, the number of optional clusters is usually unknown before the simulation (Deng et al., 2011; Liu et al., 2017). Thus, it is a big challenge to determine the cluster number when there is no prior information about cell types. Some methods are proposed to solve this problem. Rosvall and Bergstrom (2007) proposed an information-theoretic framework for resolving community structure in complex networks. The mutual information between description and network would be maximized to divide the complex network into different modules so that the number of community would be determined. Tan et al., constructed a Probability Graphic Model (PGM) framework to introduce an automatically determined function to compute the optimal number of clusters. Compared with the traditional algorithms, PGM doesn't suffer from the resolution limit and is fast enough for large data sets.

In order to obtain a relatively stable clustering result, some scientists devote to combine two or more clustering methods to get a much more accurate result (Duan et al., 2016). Ou-Yang et al.'s work (2013), they proposed a weighted ensemble clustering based on Bayesian non-negative matrix factorization (EC-BNMF) to detect the protein complexes. Recently, Yang et al. (2018) proposed an ensemble method for single cell clustering called SAFE (Yang et al., 2018). The algorithm combines the base results into a hypergraph and applies three different hypergraph partitioning algorithms to compute the final result. Moreover, Huh et al. (2020) improved this algorithm via introducing more basis clustering methods to improve the final performance named SAME. Despite considerable successes, performance of ensemble methods are still usually influenced by the base method's performance and the quite different basis clustering results will also affect the final integration results. Although EC-BNMF proposed in Ou-Yang et al. (2013) can automatically optimize the values of weights and deliver better results, the updating of iteration is time consuming. Therefore, how to tackle these limitations and design a robust emsemble clustering algorithm is still a big challenge.

In order to solve the above challenging problems, we propose an Ensemble Clustering based on Probability Graph Model with Graph Regularization (EC-PGMGR). It integrates several single-cell clustering methods in a self-regulation weighted combination form through PGM with graph regularization to produce a more accurate and informative result. In this way, EC-PGMGR can effectively reduce the limitation deriving from the base clustering results by the graph regularization to balance the relationship between the original information and the base clustering result. Besides, EC-PGMGR also can automatically determine the optimal number of the clusters through the PGM as well, which is more feasible in practical problems.

## 2. Materials and Methods

**Figure 1:**
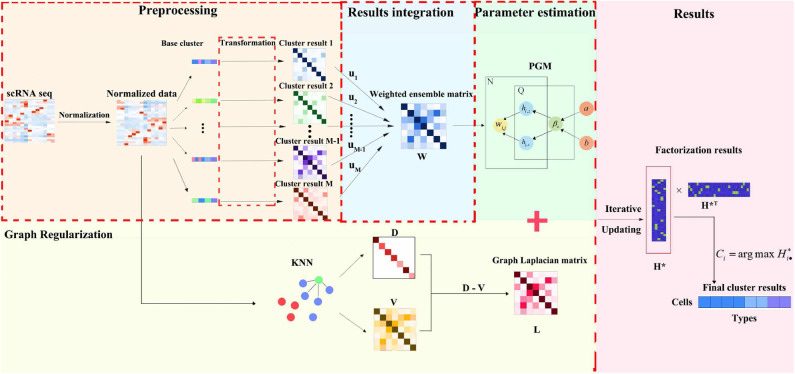
The flowchart of EC-PGMGR. It comprises of preprocessing, results integration, graph regularization and parameter estimation four steps. Normalized scRNA-seq data is first input into individual clustering methods to generate cluster results, then single results are integrated into a weight combination form, which serves as initial input to EC-PGMGR. Finally, the final clustering result can be obtained by the estimated parameters in EC-PGMGR.

We propose an ensemble clustering method consisting of four steps: data preprocessing, results integration, graph regularization and parameter estimation. Here, we briefly sketch the framework in Figure 1. Firstly, for preprocessing, the scRNA-seq data set is normalized by some classical technologies, and then dealt through some individual clustering methods. Secondly, every clustering result deriving from different base clustering algorithm is transformed into an uniform format. Then, we assemble base clustering results in a form of weight combination through a pre-learning process. After that, in order to effectively integrate clustering result from individual method and adjust the influence of the base clustering method, parameter estimation and graph regularization are employed for the integration data and the raw normalization data, respectively. Finally, via iterative updating, the final clustering result can be obtained by the estimated parameters in EC-PGMGR.

### 2.1. Data Acquisition and Preprocessing

The data exploited in this work consists of seven different single-cell data sets (Baron et al., 2016) which have “gold-standard” (deemed as true clusters) cluster labels assigned to each single cell. Baron-human1, Baron-human2, Baron-human3, and Baron-human4 are four human pancreatic islets data sets, while Baron-mouse1 and Baron-mouse2 are mouse pancreatic islets, and PBMC is human Peripheral blood mononuclear cell. Cells are barcoded by using the in-drop platform and 10X Genomics platform, respectively. The statistics of seven benchmark data sets are listed in Table 1. The preprocessing is divided into two steps. Firstly, the original scRNA-seq data is unified to be normalized by some standardized methods, such as CPM, FPKM, TPM, and etc. Secondly, the normalized expression data are considered as input data to different base clustering methods to return clustering labels.

**Table 1:** The statistics of seven benchmark data sets.

**Data sets**	**Organism**	**Single cells**	**Genes**	**True clusters**
Baron-human1	Human	1,937	16381	14
Baron-human2	Human	1,724	16381	14
Baron-human3	Human	3,605	16381	14
Baron-human4	Human	1,303	16381	14
Baron-mouse1	Mouse	822	14878	13
Baron-mouse2	Mouse	1,064	14878	13
PBMC	Human	5,132	32738	5

### 2.2. Results Integration

Suppose that we have *n* single cell data points $X={\left\{x_{1},x_{2},\ldots ,x_{n}\right\}}^{T}$, it serves as input to various base clustering methods to return a set of labels. After that, the obtained labels are transformed into an unified form so that it can assemble all these results as an initial input for EC-PGMGR. Let adjacent matrix *B*_*p*_ be the clustering result for base clustering method *p*, where the element *b*_*ij*_ equals to 1 if cell *i* and cell *j* belong to the same cluster while equals to 0 otherwise. Assume that there are *M* base clustering methods, the ensemble weighted representation *W* can be presented by Equation (1).

(1) $$\begin{array}{r} W\text{=}\overset{M}{\underset{p=1}{\sum }}u_{p}B_{p}, \end{array}$$

where *u*_*p*_ denotes the ensemble weight showing the intensity of contribution deriving from base clustering approach. In this paper, *u*_*p*_ is determined in the pre-learning process, which is calculated through internal clustering evaluation index. Firstly, we calculate the Calinski-Harabasz index *CH*_*p*_ for p-th clustering approach by Equation (2).

(2) $$\begin{array}{r} CH_{p}=\frac{\text{trace}\left(B\right)}{\text{trace}\left(W\right)}\frac{K-1}{N-K}, \end{array}$$

where $\text{trace}\left(B\right)=\overset{K}{\underset{i=1}{\sum }}||z_{i}-z|{|}^{2}$ denotes the trace of distance difference matrix between K clusters with $z_{i}=\frac{1}{\left|C_{i}\right|}\underset{x_{i}\in C_{i}}{\sum }x_{i}$ and $z=\frac{1}{N}\overset{N}{\underset{i=1}{\sum }}x_{i}$ for $N=|C_{1}|+|C_{2}|+\cdots +|C_{K}|$. Besides, $\text{trace}\left(W\right)=\overset{K}{\underset{j=1}{\sum }}\underset{x_{i}\in C_{j}}{\sum }||x_{i}-z_{j}|{|}^{2}$. Then, we normalize the *CH*_*p*_ to generate *u*_*p*_ by $u_{p}=\frac{CH_{p}}{\overset{M}{\underset{p=1}{\sum }}CH_{p}}$, such that 0 < *u*_*p*_ <1 and $\overset{M}{\underset{p=1}{\sum }}u_{p}=1$. Therefore, for an observed integrated result, *W* represents an ensemble matrix which is generated from the different base clustering results. In theory, the higher *CH*_*p*_ is, the more effective clustering performance is. So the ensemble results can effectively highlight the performance of active clustering method while weaken the performance of inactive clustering method.

### 2.3. Establishment of PGM

Note that, the element *w*_*ij*_ in *W* denotes the strength or the probability of cell *i* and cell *j* belonging to the same cell type. *W* is final represented as a binary matrix by a threshold to characterize the relationship between cell *i* and *j*. Moreover, let *h*_*iz*_ be the strength of cell *i* belonging to cell type *z*. Obviously, a higher value of *h*_*iz*_ means that cell *i* may be more like the cell type *z*. If there are *Q* types of cells (here we set *Q* is large enough, which can be sparsified by the final matrix of *H*), $\overset{Q}{\underset{z=1}{\sum }}h_{iz}h_{jz}$ means that the propensity of cell *i* and cell *j* belong to the same cell type. Hence, we assume that *w*_*ij*_ follows the Bernoulli distribution with the parameter $1-e^{-\overset{Q}{\underset{z=1}{\sum }}h_{iz}h_{jz}}$ (we use *f*(*x*) = 1−*e*^−*x*^ to make the value of latent variable $\overset{Q}{\underset{z=1}{\sum }}h_{iz}h_{jz}$ between 0 and 1). Refer to Tan and Févotte (2013), we choose automatic determination priors β_*z*_ to delete the irrelevant columns of *H* = (_*h*_*iz*_)*n* × *Q*_, where *n* represents the cell number, so that the method could set the suitable number of clusters adaptively.

(3) $$\begin{array}{r} P\left(\beta ;a,b\right)=\overset{Q}{\underset{z=1}{\prod }}P\left(\beta_{z};a,b\right)=\overset{Q}{\underset{z=1}{\prod }}\frac{b^{a}}{\Gamma \left(a\right)}\beta_{z}^{-\left(a+1\right)}exp\left(-\frac{b}{\beta_{z}}\right), \end{array}$$

where *a* and *b* are the hyperparameters. In this way, all the elements of the *z*th column of *H* would be close to zero when β_*z*_ is small, which means that this column could be deleted from the result adaptively. In order to alleviate the sensitivity of the value of β_*z*_, refer to Zhang et al. (2012), we assume that each β_*z*_ obeys an inverse Gamma distribution and independent. The PGM could be illustrated in Figure 1. By taking all these probability distributions together, one can obtain the joint probability by Equation (4).

(4) $$\begin{array}{r} P\left(W,H,\beta \right)=P\left(W;H\right)P\left(H;\beta \right)P\left(\beta ;a,b\right). \end{array}$$

### 2.4. Graph Regularization

Since the ensemble way is to integrate all the different results to a consistent one, the final result may be influenced by the base results. There would not be any better ensemble results if the base results are not good enough. Considering this problem, we use graph regularization to balance the relationship between ensemble results and original data. Similar to Deng et al. (2011), we firstly find the *K* nearest neighborhood for every cell. Specifically, if cell *i* is one of the KNN of cell *j* or cell *j* is the KNN of cell *i*, we set *v*_*ij*_ = *v*_*ji*_ = 1, while *v*_*ij*_ = 0 otherwise. Secondly, we compute the degree $d_{i}=\overset{n}{\underset{i=1}{\sum }}v_{ij}$, and generate a diagnose matrix *D* = (*d*_*ij*_) = *diag*(*d*_1_, *d*_2_, ..., *d*_*n*_). Thirdly, we define the graphical Laplacian matrix *L* = *D*−*V*. Then the graph regularization term is written by Equation (5)

(5) $$\begin{array}{r} R_{1}=\frac{1}{2}\overset{n}{\underset{i=1}{\sum }}\overset{n}{\underset{j=1}{\sum }}{\left(h_{iz}-h_{jz}\right)}^{2}v_{ij}. \end{array}$$

Finally, we obtain the optimal result by minimizing the objective function with the regularization term *R*_1_ by Equation (6).

(6) $$\begin{array}{l} \underset{U,H,\beta }{min}(-logP(W,H,\beta )+\alpha R_{1})\\ =\underset{U,H,\beta }{min}(-logP(W;H)-logP(H;\beta )-logP(\beta ;a,b)+\alpha R_{1}), \end{array}$$

here α ∈ IR is the regularization parameter. The details of the construction of PGM and the graph regularization are listed in Supplementary Equations (1)–(8).

### 2.5. Parameters Estimation

In order to obtain the final results, we need to update *H* and β alternately. Similar to the solution way in Ou-Yang et al. (2013), we use the multiplicative updating rules to get *H* = (*h*_*iz*_) and β = (β_*z*_) described in Equations (7) and (8), respectively.

(7) $$\begin{array}{r} h_{iz}\leftarrow \frac{1}{2}h_{iz}+\frac{1}{2}h_{iz}\times \frac{\overset{n}{\underset{j=1}{\sum }}w_{ij}\frac{1}{1-\exp\left(-\overset{Q}{\underset{z=1}{\sum }}h_{iz}h_{jz}\right)}h_{jz}+\alpha \overset{n}{\underset{j=1}{\sum }}v_{ij}h_{jz}}{\overset{n}{\underset{j=1}{\sum }}h_{jz}+\frac{h_{iz}}{2\beta_{z}}+\alpha \overset{n}{\underset{j=1}{\sum }}d_{ij}h_{jz}}, \end{array}$$

and

(8) $$\begin{array}{r} \beta_{z}\leftarrow \frac{2b+\sum_{i=1}^{n}h_{iz}^{2}}{n+2a+2}. \end{array}$$

To sum up, we iteratively update *H*, β above until they satisfy a stopping criterion. Let β_new_ and β_old_ be the vector of β at the current and previous iterations, respectively. The algorithm is stopped whenever ||β_new_−β_old_||_*F*_ < ρ, Here, we set the value of ρ to be 1*e*−5. Furthermore, we limit the calculation procedure to a maximum of 100 iterations for practical purposes. That is, we stop iterating when ||β_new_−β_old_||_*F*_ < ρ or the number of iterations reach 100. In order to avoid a local minimum, we repeat the algorithm 50 times with random initial input of *H* and choose the result that outputs the lowest value of objective function (7).

### 2.6. Final Clusters Determination

By using the update way described above, we could get the clustering matrix H^*^ which is the final updating result of *H*. We obtain the optimal number of clusters *C* which represent the number of columns of the biggest element. The details of EC-PGMGR are presented in Algorithm 1.

**Algorithm 1:** Ensemble Clustering based on Probability Graphic Model with Graph Regularization (EC-PGMGR).

Input: **X**: scRNA-seq data; **Q**: Maximum number of cluster cell types; **a, b**: Hyperparameters related to β distribution function; **ρ**: Iteration stop threshold; **α**: Graph regularization term parameter.
Output: H^*^: Final cluster matrix; **C**: Cell types.
1: Input sc-RNA seq data into base clustering methods to generate cluster labels and calculate *CH* index by Equation (2) and get weight by the normalized way described in section 2.2.
2: Construct the transform matrix *B*_*p*_ (*p* = 1, 2, ..., *M*) respectively and calculate the observed matrix *W* by Equation (1).
3: Using the KNN on the original data to obtain every single cell's *K* nearest neighborhood to compute the KNN neighborhood matrix *V* and the Laplacian matrix *L* = *D*−*V*.
4: Initialize base matrix *H*.
5: Update *H* and β according to iterative update rule Equations (6) and (7).
6: Calculate the value of objective function Equation (3) (The detailed objective function is listed in Supplementary Equation 8.
7: Repeat step 4 and step 5 until the iteration stop rules are achieved.
8: Return *H* as H^*^.
9: Calculate $C_{i}=argmaxH_{i.}^{*}$ and obtain *C*.

## 3. Experimental Results and Discussion

In order to validate the performance of EC-PGMGR, the comparative experiments are employed on seven benchmark data sets through SAFE R package (Yang et al., 2018). All calculations and simulations are carried out by using MATLAB on a PC with 3.2G Hz Intel Core i5 CPU, 8GB RAM, and windows 10 64-bit ultimately.

### 3.1. Evaluation Metric

Since all these data sets have the gold-standard labels, we choose the Adjusted Rand Index (ARI) and Normalized Mutual information (NMI) as the evaluation index to measure the performance. The definitions are presented by Equations (9) and (10), respectively.

(9) $$\begin{array}{r} ARI=\frac{\sum_{i,j}\left(\begin{array}{c} n_{ij}\\ 2 \end{array}\right)-[\sum_{i}\left(\begin{array}{c} a_{i}\\ 2 \end{array}\right)\sum_{j}\left(\begin{array}{c} b_{j}\\ 2 \end{array}\right)]/\left(\begin{array}{c} n\\ 2 \end{array}\right)}{\frac{1}{2}[\sum_{i}\left(\begin{array}{c} a_{i}\\ 2 \end{array}\right)+\sum_{j}\left(\begin{array}{c} b_{j}\\ 2 \end{array}\right)]-[\sum_{i}\left(\begin{array}{c} a_{i}\\ 2 \end{array}\right)\sum_{j}\left(\begin{array}{c} b_{j}\\ 2 \end{array}\right)]/\left(\begin{array}{c} n\\ 2 \end{array}\right)}, \end{array}$$

(10) $$\begin{array}{r} NMI=\frac{2\times \sum_{i,j}p_{ij}\log\frac{p_{ij}}{p_{i}\times p_{j}}}{-\sum_{i}p_{i}\log p_{i}-\sum_{j}p_{j}\log p_{j}}, \end{array}$$

where *n* is the total number of cell; *a*_*i*_ and *b*_*j*_ represent the number of cells in estimated cluster *i* and in true cluster *j*, and *n*_*ij*_ is the number of cells shared by estimated cluster *i* and true cluster *j*. Besides, *p*_*ij*_ = *n*_*ij*_/*n*, *p*_*i*_ = *a*_*i*_/*n* and *p*_*j*_ = *b*_*j*_/*n*. Both ARI and NMI range from 0 to 1, higher the scores reflect the effective and informative clustering result.

### 3.2. Model Specification

In this section, we introduce the settings of normalization method, individual clustering method for preprocessing and the settings of parameters in the new proposed model.

#### 3.2.1. Initial Settings in Preprocessing

Counts Per Million mapped reads (CPM) is used as a normalized method, reads count are divided by the total reads count and the result is multiplied by 1,000,000. Thus, the prepared scRNA-seq data sets are considered as input to four (*p* = 4 but not limited to that) commonly-used individual clustering methods SC3 (Kiselev et al., 2017), CIDR (Li et al., 2017), Seurat (Satija et al., 2015), and t-SNE+k-means (Dominic et al., 2015). The results generated by these base methods are shown in Table 2.

**Table 2:** The number of clusters generated by different methods.

**Data sets**	**True clusters**	**SC3**	**CIDR**	**Seurat**	**t-SNE+k-means**	**SAFE**	**SAME**	**EC-PGMGR**
Baron-human1	14	20	8	11	10	15	9	11
Baron-human2	14	18	7	10	8	7	7	8
Baron-human3	14	31	4	13	8	8	8	7
Baron-human4	14	14	6	9	5	5	7	6
Baron-mouse1	13	15	13	9	4	5	6	9
Baron-mouse2	13	15	6	9	12	14	8	9
PBMC	5	361	8	12	8	349	4	7

#### 3.2.2. Initial Settings in EC-PGMGR

In EC-PGMGR, there are five parameters *Q*, *a*, *b*, α and *W*_*t*_ (threshold to filter *W*) need to be predefined. *Q* is the initial setting of number of cell type, which can be shrinked through other settings. Note that the true cluster number ranges from 5 to 14 of our experimental data sets, here we set *Q* to be 25. Besides, we set *W*_*t*_ to be 0.5, which means that two cells are classified into on category (*w*_*ij*_ = 1) if more than half of the methods considering the two cells to be in a class. Observing that the shape hyperparameter *a* affects the optimization of the objective function Equation (6) only through the updating rule Equation (8), thus the influence of *a* is moderated by the number of nodes *n*. Therefore, we fix *a* = 1 and vary the value of *b* to find the best result for each data set. Another key parameter is α which control the effect of graphical regularization term *R*_1_, the model is degraded into EC-PGM when α = 0. Finally, the key parameters that affect the performance of EC-PGMGR are *b* and α.

**Figure 2:**
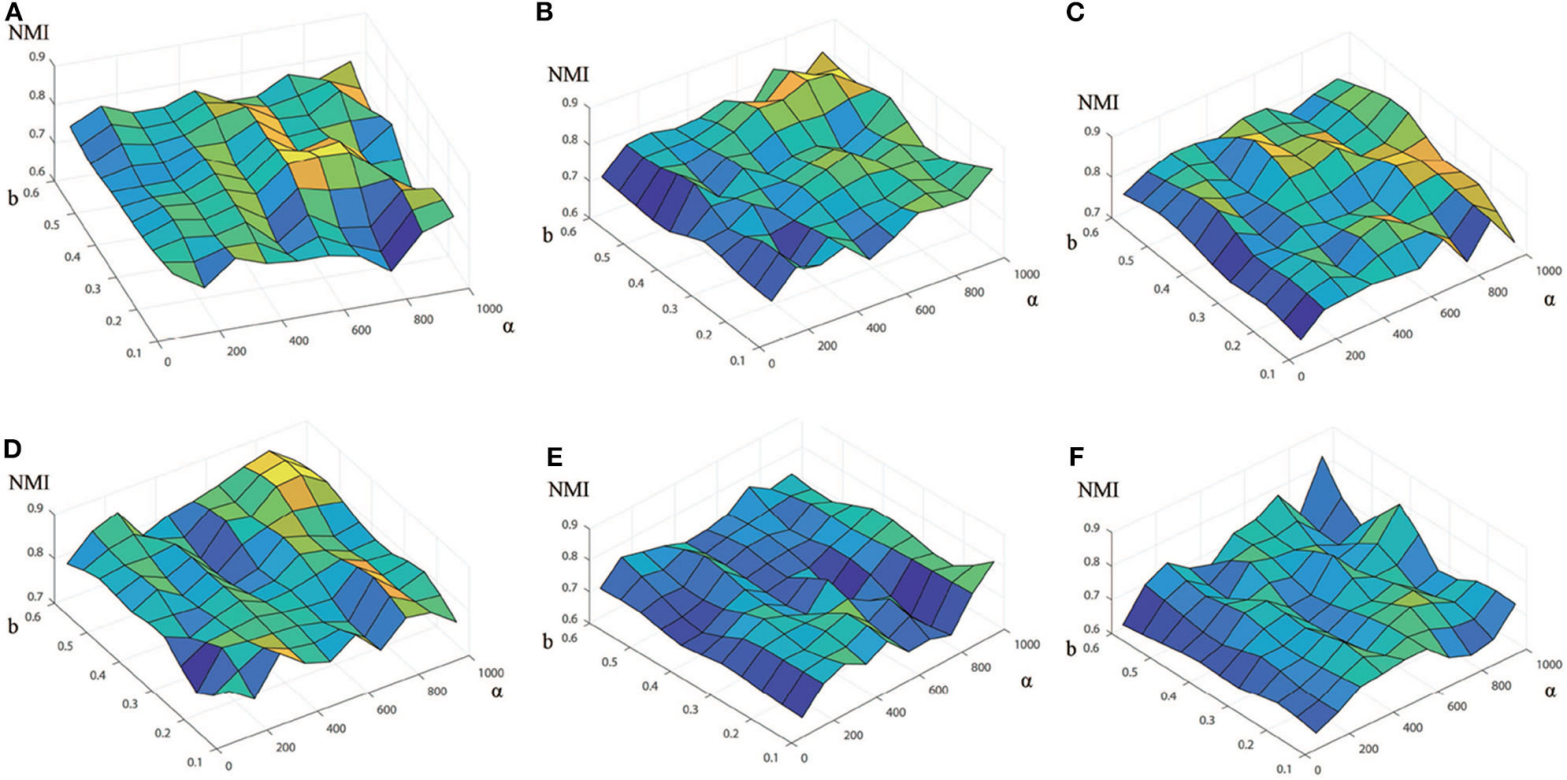
Performance of EC-PGMGR on 6 benchmark data sets with respect to different values of *b* and α measured in terms of the NMI score. The x-axis denotes the value of α, the y-axis denotes the value of *b*, and the z-axis denotes the value of the NMI. **(A)** Baron-human1. **(B)** Baron-human2. **(C)** Baron-human3. **(D)** Baron-human4. **(E)** Baron-mouse1. **(F)** Baron-mouse2.

In order to fully understand how these two parameters affects the performance, we vary the values of *b* and α for each data set, and compare the performances in terms of NMI (Figure 2) and ARI (Supplementary Figure 2) with respect to two reference sets. For each data set, the pair of parameters is grid-searched in the range of *b* (*b* ∈ {0.1, 0.2, ⋯ , 0.6}) and α (α ∈ {50, 150, 250, 350, ⋯ , 950}). As shown in Figure 2, for a fixed value of α, the NMI increases initially and decrease after reaching the maximum as the value of *b* increases, and this is true for all the data sets. Thus, we can find from Figure 2 that the optimal result are obtained when *b* = 0.3 and α= 650 for Baron-human1, *b* = 0.5 and α = 750 for Baron-human2, *b* = 0.4 and α = 950 for Baron-human3, *b* = 0.5 and α = 850 for Baron-human4, *b* = 0.1 and α = 950 for Baron-mouse1 and *b* = 0.4 and α = 950 for Baron-mouse2. In the following, unless otherwise stated, the final results are obtained with these optimal values of parameters for the 6 data sets.

### 3.3. Results and Analysis

We carry out some comparative experiments on six data sets to validate the performance of EC-PGMGR in terms of effectiveness, robustness with the variation of cluster number *Q* and the basis method. Besides, some biological explanations are presented to evaluate the rationality of the new proposed model.

#### 3.3.1. Robustness of Selection of Initial Setting Cluster Number *Q*

**Figure 3:**
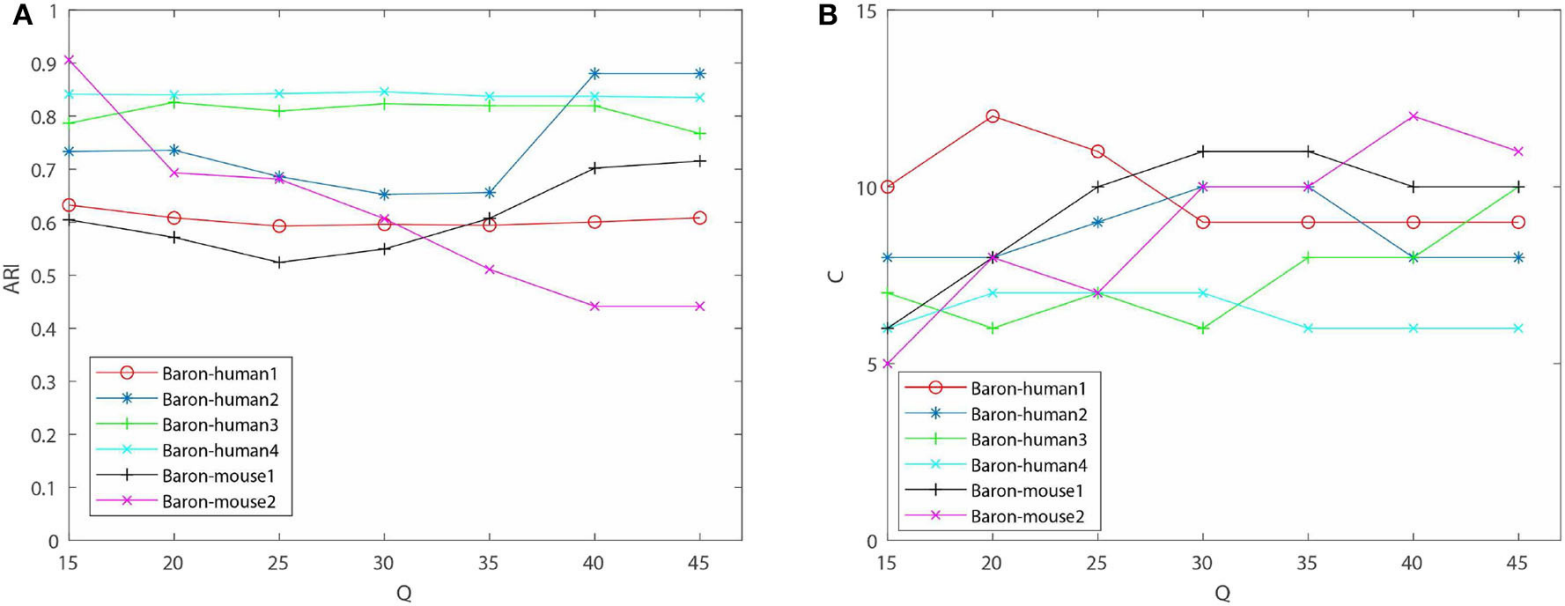
Performance of EC-PGMGR on 6 benchmark data sets with *Q*. **(A)** Results on ARI of EC-PGMGR on 6 benchmark data sets with *Q* ranges from 15 to 45. **(B)** Results of final cluster number *C* on 6 benchmark data sets with *Q* ranges from 15 to 45.

One of contributions of EC-PGMGR is that the cluster result is not influenced by the initial setting of the cluster number. Note that, *Q* denotes the initial setting of the cluster number, and *C* denotes the type of final cluster result calculated by using EC-PGMGR. As shown in Figure 3A and Table 2, the variations of ARI are gentle on data sets except of Baron-human2 and Baron-mouse 2 with the *Q* ranging from 15 to 45 with 5 step. Besides, as shown in Figure 3B, the final cluster number *C* is stable at a little range from 7 to 11 although the initial setting *Q* ranges from 15 to 45 with 5 step, as well. Therefore, the experiment proves that our EC-PGMGR can automatically calculate the optimal cluster result which does not depend on the setting of the initial value of *Q*.

#### 3.3.2. Robustness of Selection of Basis Clustering Algorithm

The aim of ensemble clustering is to improve the stability, robustness, and accuracy of the final results by integrating multiple clustering results. The limitation of many ensemble clustering is that the final cluster result depends on the selection of the basis cluster methods. In order to validate the robustness of EC-PGMGR, we do some comparative experiments from two aspects: (1) Consider various number of base cluster methods. (2) Adjust the results of base cluster method.

**Figure 4:**
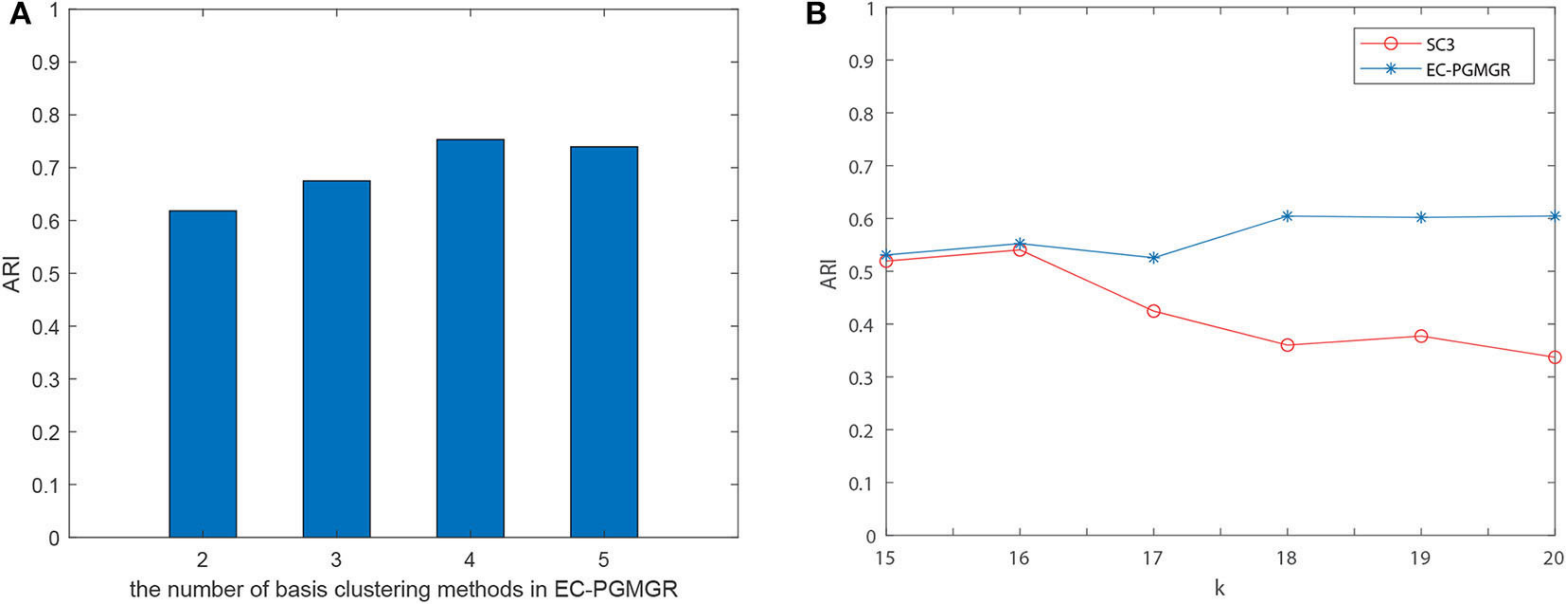
**(A)** The performance of different combination of basis cluster on Baron-mouse1. **(B)** Assessing the robustness of EC-PGMGR when varying the number of clusters for SC3 results and holding the other three individual methods constant in Baron-human1. Different *k* will influence the results of SC3.

Ref to SAME (Huh et al., 2020), we firstly select the number of base cluster method, and calculate the results for different clustering method. As Figure 4A depicted, we find that the clustering performance doesn't change too much when the number of base clustering methods increasing. For example, in Baron-mouse1, we choose the best basis clustering method as each combination of ensemble clustering with EC-PGMGR and the result stays stable when the number of basis methods increases. Secondly, because it is convenient to adjust the results of method of SC3 by setting different parameters, we choose to take the experiments on all data sets and set the initial cluster number *k* (*k* from 15 to 20 with 1 step size for Baron-human1, Baron-human2, Baron-human3, Baron-human4; *k* from 10 to 15 with 1 step size for Baron-mouse1, Baron-mouse2) for SC3 clustering method to observe whether the difference generated by SC3 would affect the results of EC-PGMGR. Figure 4B shows the performance of EC-PGMGR when base cluster result (SC3) changes on Baron-human1. The other results on the other data sets are shown in Supplementary Figure 3. From the results, we can see that our EC-PGMGR could be well-stabilized with a good performance. Even when the results of SC3 method changes, EC-PGMGR's performance is still better than that of SC3. The algorithm could balance base clustering results and original data through graph Laplacian regularization to keep robust.

#### 3.3.3. Effectiveness of EC-PGMGR

**Figure 5:**
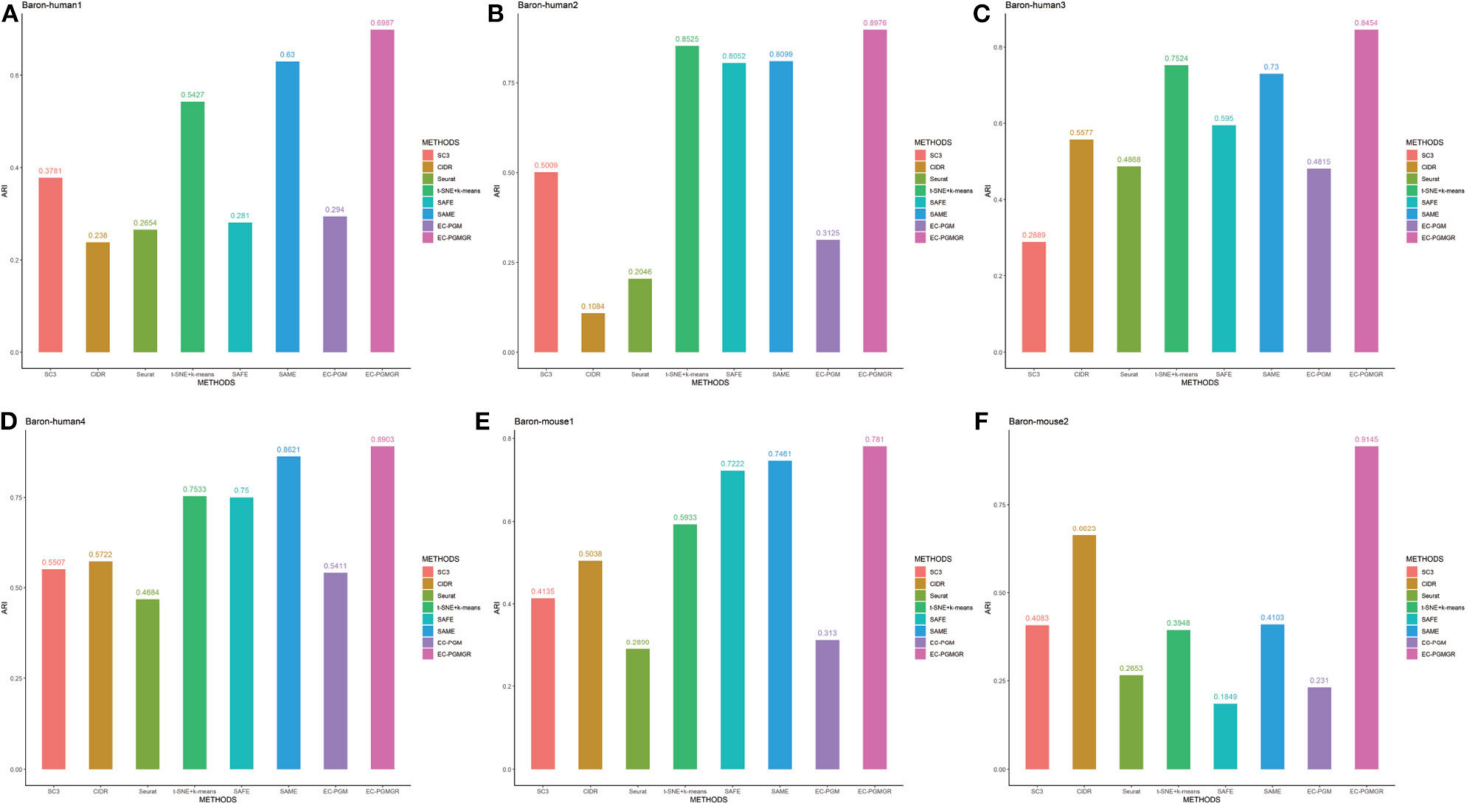
Performance on different data sets with different methods in terms of ARI. SC3, CIDR, Seurat and t-SNE+k-means are the individual methods, while SAFE, SAME, EC-PGM and EC-PGMGR are the ensemble clustering methods. **(A)** Baron-human1. **(B)** Baron-human2. **(C)** Baron-human3. **(D)** Baron-human4. **(E)** Baron-mouse1. **(F)** Baron-mouse2.

**Figure 6:**
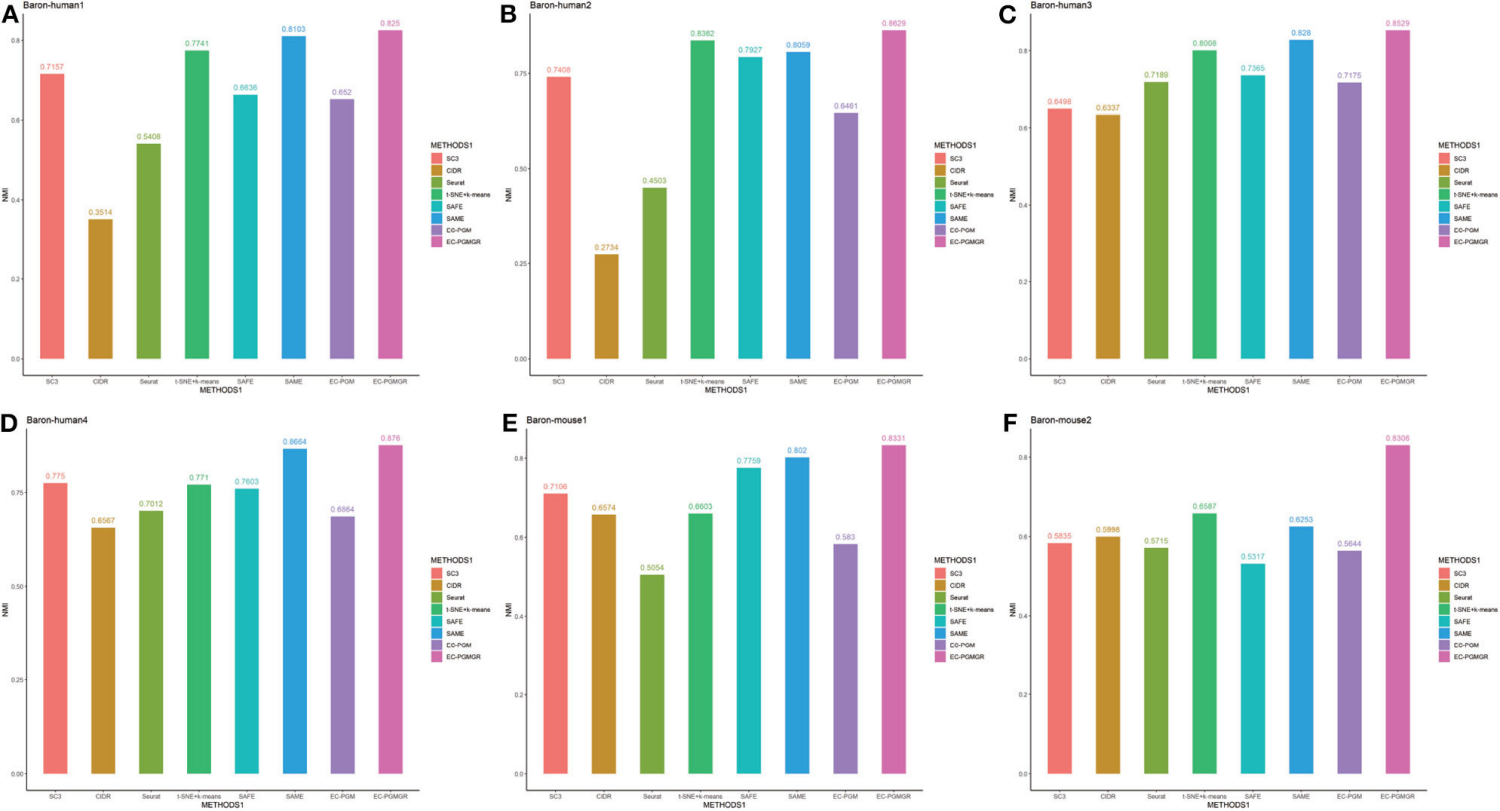
Performance on different data sets with different methods in term of NMI. SC3, CIDR, Seurat and t-SNE+k-means are the individual methods, while SAFE, SAME, EC-PGM and EC-PGMGR are the ensemble clustering methods. **(A)** Baron-human1. **(B)** Baron-human2. **(C)** Baron-human3. **(D)** Baron-human4. **(E)** Baron-mouse1. **(F)** Baron-mouse2.

**Figure 7:**
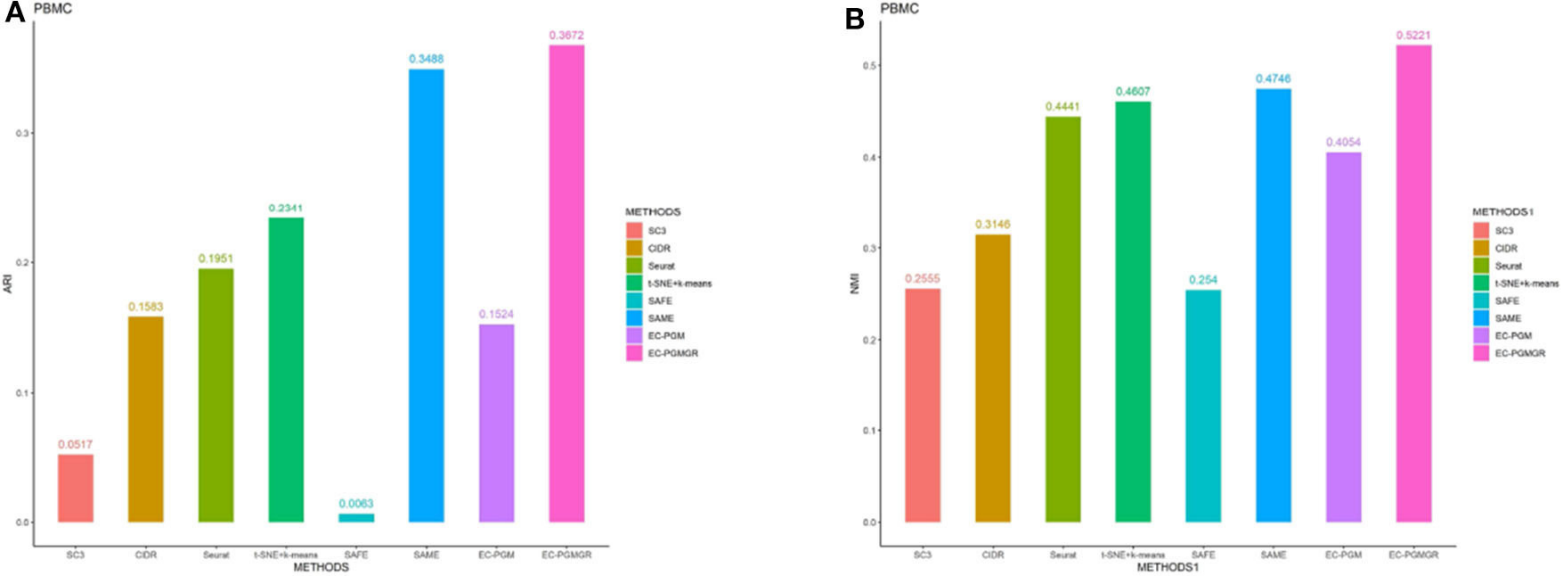
The performance with different methods on PBMC data set. **(A)** The ARI performance. **(B)** The NMI performance.

In order to validate the effectiveness of EC-PGMGR, we compare our method with individual clustering methods SC3, CIDR, Seurat, t-SNE+*k*-means and two ensemble clustering methods SAFE (Yang et al., 2018) and SAME (Huh et al., 2020) in terms of ARI and NMI. As Figures 5, 6 shown, our new proposed EC-PGMGR (8th method) can achieve good performance on different data sets. The base results are generated by the function in the SAFE clustering method with its default parameters and these base clustering results are used in SAFE, SAME, EC-PGM, and EC-PGMGR to generate an ensemble result, where EC-PGM is degraded model with non-regularization of EC-PGMGR. The final results validate the effectiveness of the process of regularization. In the case of non-regularization, the average ARI and NMI of 6 data sets are about 0.33 and 0.56, and then they increase to 0.83 and 0.84, respectively. Besides, we also apply the different methods on a large PBMC data set. The comparison results are presented in Figure 7. We can see that the SAFE method performs not well on PBMC data set while the performance difference between SAME and EC-PGMGR is small. We further count the clustering results deriving from different clustering methods on the PBMC data set shown in Table 2. We find that the result of SC3 method is not very well. With the default parameters, the SC3 method divide cells into 361 clusters and this will influence the performance of SAFE. With these setting, we find that even the performance of base clustering result is not well, our method can still achieve good performance.

#### 3.3.4. Other Analysis

**Figure 8:**
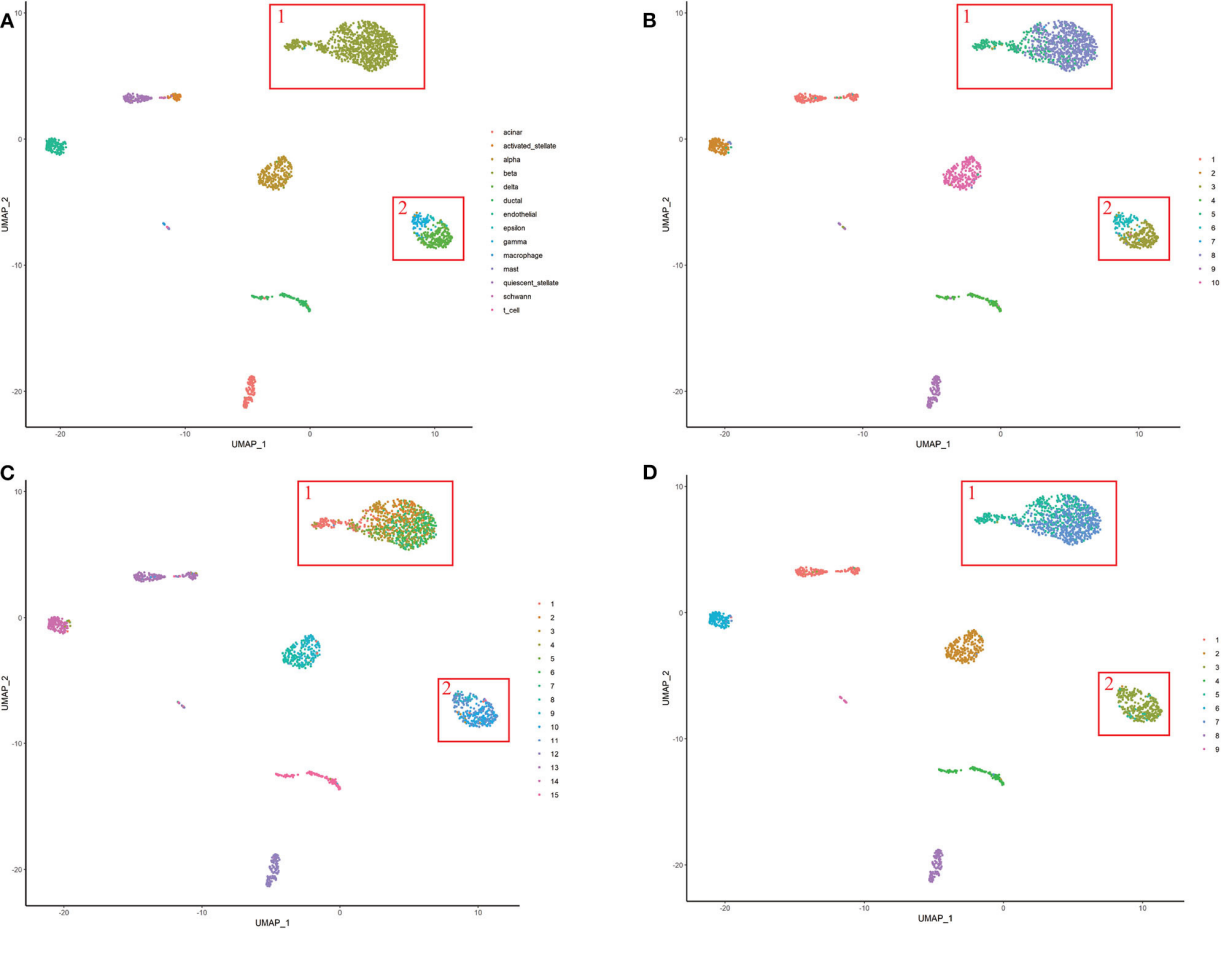
The visualization of performance of different methods on Baron-human1. **(A)** True label. **(B)** EC-PGMGR. **(C)** SAFE. **(D)** SAME.

**Figure 9:**
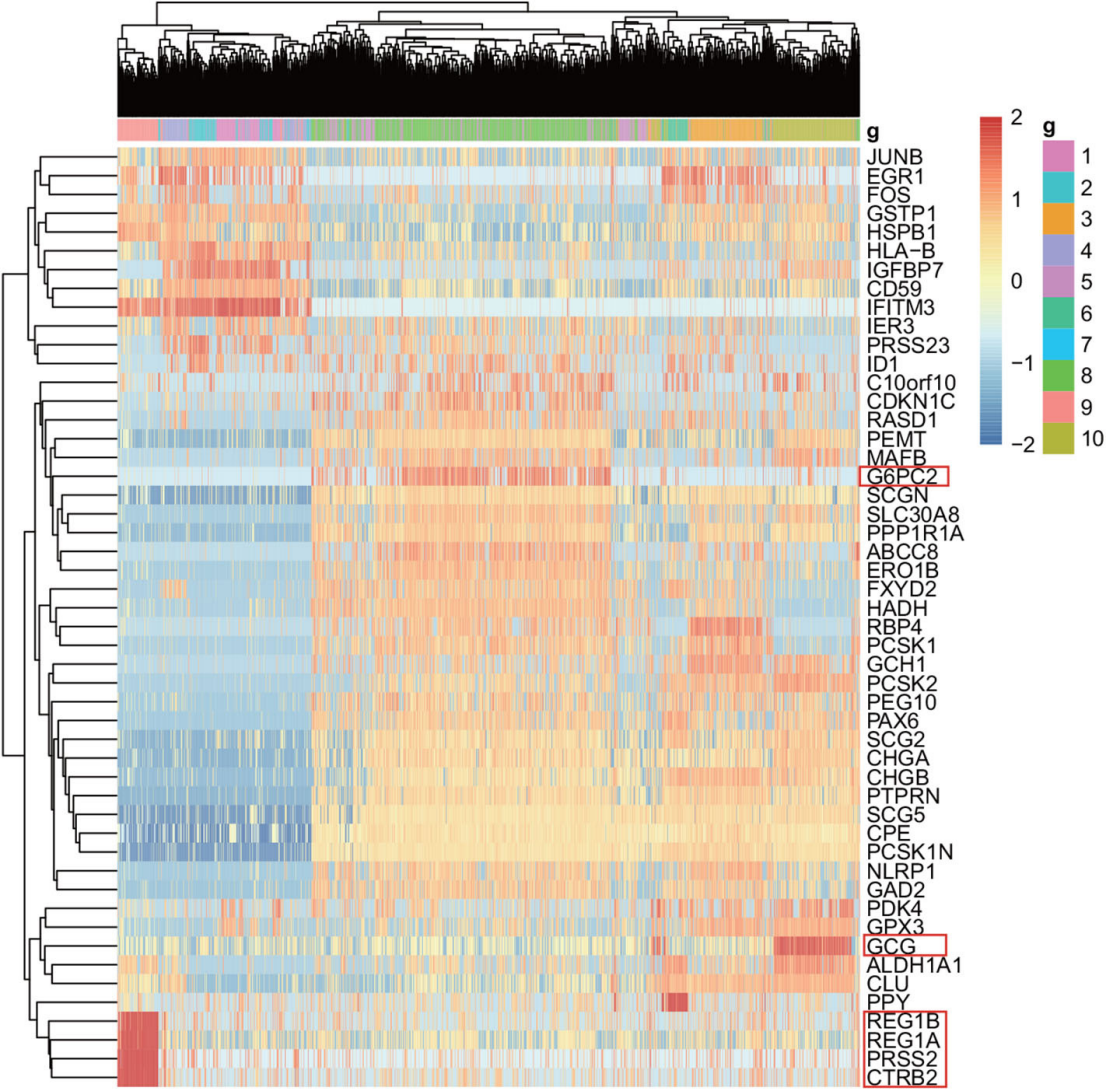
The heat map shows the top 50 standard deviation genes in the Baron-human1 experimental results. Each row represents the genes and each column represents the cells.

For the purpose of the evaluation of the biological significance, we do some correlation analysis among some marker genes of cells. We use Unified Manifold Approximation and Projection (UMAP) (McInnes and Healy, 2018), which is a new dimensionality reduction manifold learning technology to visualize the results of the Baron-human1. In terms of visual quality, the UMAP algorithm has a competitive advantage with t-SNE, but it retains more global structure, superior operating performance, and better scalability. As shown in Figure 8, the visualization of three ensemble methods and the true label. We can see that our method can achieve better result than other two methods in Baron-human1. We see that all three ensemble methods can achieve good clustering performance according to the true label. In area 1, the true clustering result is defined as beta cells, EC-PGMGR and SAME method can divided well. And in area 2, the result which generated by EC-PGMGR is closet to the real type. Besides, Figure 9 shows the heat map of the top 50 standard deviation genes in the Baron-human1 experimental results. It can be seen that there are clearly high-expressed genes in the results we gathered. We queried some of these highly expressed genes and found that REG1B, REG1A, PRSS2, CTRB2 belong to Acinar cell's marker genes. GCG is the marker gene of Alpha cell and G6PC3 belongs to Beta cell's markers (Li et al., 2016). The results illustrates that our method achieves a good performance in clustering. The other experiments are listed in Supplementary Figures 4, 5, respectively.

## 4. Conclusion

We propose EC-PGMGR algorithm, an unsupervised ensemble clustering method using PGM with graph Laplacian regularization. Unlike conventional ensemble clustering algorithms that treat each base clustering result equally, EC-PGMGR is a weighted ensemble clustering algorithm which can automatically equip with weights for different base clustering results by a pre-learning process. Therefore, base clustering results that obtain higher weights may be more reliable and can be regarded as active clustering method. On the contrary, base clustering results with lower weights may be less reliable and they may be far away from real cases. Our EC-PGMGR method can integrate different kinds of single cell clustering results and obtain an optimal consensus clustering results. Considering that the proper single cell clustering algorithm require the number of clusters, EC-PGMGR can effectively and adaptively optimizing the number of clusters, which is more reasonable for practical scientific research. To avoid the undesirable ensemble result which could be caused by the base clustering results, graph Laplacian regularization is used in EC-PGMGR to preserve the information of original data, which can balance the base results and the original information to reduce the effect deriving from some inactive base clustering results.

We take experiments on seven single-cell data sets which have different sizes, species, and platforms. The ARI and NMI show that our method is better than the other comparative methods including individual and ensemble clustering methods on different data sets. We find that the some experimental performances on Baron-mouse2 are always not satisfying. Considering that the data type we use is in-drop data, there are many zeros in its expression matrix due to some technical reasons. Although part of zero data is the true expression of cells, there is still some data which doesn't reflect the real expression level (van Dijk David et al., 2018; Svensson et al., 2017). The zero-inflated data will influence the final clustering results since the data is partly inaccurate. We calculate the ratio of 0 values in each data set to find out if there are relationships between the data and the not good performances. Results shows that the ratio of 0 in Baron-human4 and Baron-mouse2 is higher than the others (Baron-human1: 0.096; Baron-human2: 0.0949; Baron-human3: 0.0978; Baron-human4: 0.1100; Baron-mouse1: 0.0952; Baron-mouse2: 0.1220). It may explain why the performance is not very good on Baron-mouse2. Too much missing in the original data will influence the base cluster results and graph regularization term. The further researches would integrate more single-cell clustering methods and perform preliminary screening for base clustering methods, and then perform integrated analysis. The missing scRNA-seq data should be filled first so that the downstream analysis could be more accurate and reasonable. Besides, we estimate the overall time cost of the updating process in Equations (7) and (8). The time cost for updating *H* is *O*(*n*^2^*Q*), where *n* is the number of cells, and *Q* is the number of initial clusters. The time cost for updating β is *O*(*nQ*). Therefore the overall time cost of EC-PGMGR is *O*(*n*^2^*QT*), where *T* is the number of iterations. Since the parameter *H* is sparse, the real time cost is much smaller than *O*(*n*^2^*QT*). In addition, before performing our ensemble algorithm, we need to compute Laplacian matrix *L* which is time consuming. It can be improved by some computational techniques in the further study. As an ensemble clustering algorithm, our model is more flexible. It is of great interest to use this model to undertake other clustering-based tasks such as exploring modules in gene regulatory networks and cell signaling networks.

## Data Availability Statement

A MATLAB package for the EC-PGMGR algorithm and the six data sets can be available through github (https://github.com/zhuyuan-cug/zhuyuan-lab).

## Author Contributions

YZ and D-XZ conceived and designed the work and wrote the original manuscript. D-XZ carried out computer implementation and data analysis. X-FZ and LO-Y interpreted and checked the simulation results. MY supervised the project. MY, X-FZ, LO-Y, and MW contributed to the writing of final manuscript. All authors contributed to the article and approved the submitted version.

## Conflict of Interest

The authors declare that the research was conducted in the absence of any commercial or financial relationships that could be construed as a potential conflict of interest.
