# Supplementary material for: EC-PGMGR: Ensemble Clustering Based on Probability Graphical Model With Graph Regularization for Single-Cell RNA-seq Data
Source: Front Genet. 2020 Nov 4;11:572242. doi: 10.3389/fgene.2020.572242 (PMC7673820; doi:10.3389/fgene.2020.572242)
Supplement: Supplementary file 1 [file Data_Sheet_1.PDF]

Supplementary Material

1 DETAILS FOR EC-PGMGR

In this file, we will introduce the construction of PGM and regularization in detailed. Supposed that there are Q types of cells, we use a latent variable h_{iz} to represent the strength of cell i's membership of cell type z. A higher value of h_{iz} means that cell i may be more like the cell type z. And the value of $\sum_{z=1}^{Q} h_{iz}h_{jz}$ means the propensity of cell i and cell j belong to the same cell type. Thus, elements w_{ij} in W which represents the relationship between cell i and cell j, follows the Bernoulli distribution with the parameter $\sum_{z=1}^{Q} h_{iz}h_{jz}$. We can obtain the probability

$$P(W;H) = \prod_{i,j=1}^{n} (\sum_{z=1}^{Q} h_{iz}h_{jz})^{w_{ij}} (1 - \sum_{z=1}^{Q} h_{iz}h_{jz})^{1-w_{ij}}. \tag{S1}$$

In order to ensure that the value of latent variable is between 0 and 1, here we make some rewrites of the probability model

$$P(W;H) = \prod_{i,j=1}^{n} (1 - \exp(-\sum_{z=1}^{Q} h_{iz}h_{jz}))^{w_{ij}} (\exp(-\sum_{z=1}^{Q} h_{iz}h_{jz}))^{1-w_{ij}}. \tag{S2}$$

While in practice, the parameter Q (the number of clusters) is unknown initially. Generally, Q is set to a large enough value, but it will lead to high computational complexity. Here, refer to (Tan *et al.* 2013), we choose automatic determination priors β_z to delete the irrelevant columns of $H(h_{iz})$ so that the method could adaptive compute the best number of clusters. Therefore, we set the variance β_z as:

$$P(h_{iz};\beta_z) = HN(h_{iz};\beta_z), \tag{S3}$$

where for $h_{iz} \geq 0$, $HN(x;\beta_z) = (\frac{2}{\pi\beta_z})^{\frac{1}{2}} exp(-\frac{x^2}{2\beta_z})$, $P(H;\beta) = \prod_{i=1}^{n}\prod_{z=1}^{Q} HN(h_{iz};\beta_z)$, and otherwise, $HN(h_{iz};\beta_z) = 0$. By this way, all the elements of the zth column of H would be close to zero, when β_z is small, which means this column could be delete from the result adaptively. In order to alleviate the sensitivity of the value of β_z, similar to (Zhang *et al.* 2012), we assume that each β_z obeys an inverse Gamma distribution and independent. So the final joint distribution of $\beta = [\beta_z]$ is

$$P(\beta;a,b) = \prod_{z=1}^{Q} P(\beta_z;a,b) = \prod_{z=1}^{Q} \frac{b^a}{\Gamma(a)} \beta_z^{-(a+1)} exp(-\frac{b}{\beta_z}), \tag{S4}$$

where a and b are the hyperparameters, respectively. Considering of all these factors, the PGM could be written as follows,

$$P(W,H,\beta) = P(W;H)P(H;\beta)P(\beta). \tag{S5}$$

With model S5, we will maximum the joint probability to estimate the values of H and β. By taking all these probability distribution together, we could obtain the objective function as follows.

$$\begin{aligned}
&\min_{U,H,\beta} -logP(W,H,\beta) \\
&= -logP(W;H) - logP(H;\beta) - logP(\beta) \\
&= -\sum_{i=1}^{n}\sum_{j=1}^{n}(w_{ij}log(1-exp(-\sum_{z=1}^{Q}h_{iz}h_{jz})) \\
&-(1-w_{ij})\sum_{z=1}^{Q}h_{iz}h_{jz} + \sum_{i=1}^{n}\sum_{z=1}^{Q}\frac{1}{2\beta_z}(h_{iz})^2 \\
&+\frac{n}{2}\sum_{z=1}^{Q}log\beta_z + \sum_{z=1}^{Q}\frac{b}{\beta_z} + (a+1)\sum_{z=1}^{Q}log\beta_z \\
&s.t.\ H>0, and \sum_{p=1}^{M}u_p = 1,\ u_p \geq 0\ for\ p=1,2,...,M.
\end{aligned} \tag{S6}$$

Here H represents the cell type relationship matrix. However, since the ensemble way is to integrate all the different results to a consistent one, the final result will be influenced by the basis results. If the basis results are all wrong, the ensemble result can not be better. Considering this problem, we use graph regularization to balance the relationship between ensemble results and original data. Firstly, we find the K nearest neighborhood for every cells. Detailed, if cell i is one of the KNN of cell j or cell j is the neighborhood of cell i, we set $v_{ij} = v_{ji} = 1$, while $v_{ij} = 0$ otherwise. Secondly, we compute the degree $d_i = \sum_{i=1}^{n} v_{ij}$, and generate a diagnose matrix $D = diag(d_1, d_2, ..., d_n)$. Thirdly, we define the graphical Laplacian matrix $L = D - V$. So the graph regularization term could be written as follows,

$$\begin{aligned}
&R_1 = \frac{1}{2}\sum_{i=1}^{n}\sum_{j=1}^{n}(h_{iz}-h_{jz})^2 v_{ij} = Tr(H^T LH) \\
&= \sum_{i=1}^{n}\sum_{j=1}^{n}[l_{ij}\sum_{z=1}^{Q}h_{iz}h_{jz}] \\
&= \sum_{i=1}^{n}\sum_{j=1}^{n}\sum_{z=1}^{Q}l_{ij}h_{iz}h_{jz} = \sum_{i=1}^{n}\sum_{j=1}^{n}\sum_{z=1}^{Q}(d_{ij}-v_{ij})h_{iz}h_{jz}.
\end{aligned} \tag{S7}$$

In practice, the chosen of different K in KNN will produce different results. There is currently no theoretical method for the choice of K value. And we set the value of K from 2 to 5 with step 1 on different data sets. As the **Supplementary Fig.1** shown, the performance of most data sets is declined or stayed when the K value is greater than 4, and for the speed of calculation, we finally choose 4 as the K value in our later experiment. Finally, we obtain the optimal result by minimizing the objective function with the regularization term $R_1 = Tr(H^T LH) = \sum_{i=1}^{n}\sum_{j=1}^{n}\sum_{z=1}^{Q} l_{ij}h_{iz}h_{jz}$ as the equation (S8).

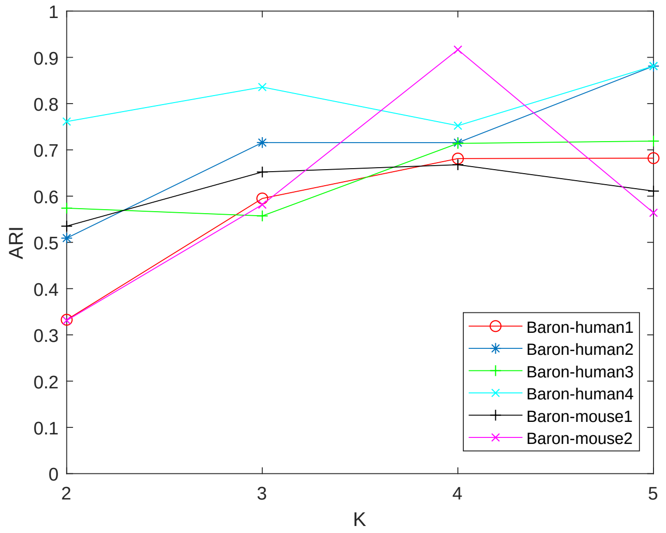

Figure S1. The influence of Different construction of graph Laplacian matrix to the EC-PGMGR. The Effect of different chosen of K nearest neighbour.

$$
\begin{aligned}
&\min_{U,H,\beta}(-logP(W,H,\beta)+\alpha R_1)\\
&=-logP(W;H)-logP(H;\beta)-logP(\beta)+\alpha R_1\\
&=-\sum_{i=1}^{n}\sum_{j=1}^{n}(w_{ij}log(1-exp(-\sum_{z=1}^{Q}h_{iz}h_{jz}))\\
&-(1-w_{ij})\sum_{z=1}^{Q}h_{iz}h_{jz}+\frac{n}{2}\sum_{z=1}^{Q}log\beta_z\\
&+\sum_{i=1}^{n}\sum_{z=1}^{Q}\frac{1}{2\beta_z}(h_{iz})^2+\sum_{z=1}^{Q}\frac{b}{\beta_z}\\
&+(a+1)\sum_{z=1}^{Q}log\beta_z+\alpha\sum_{i=1}^{n}\sum_{j=1}^{N}\sum_{z=1}^{Q}l_{ij}h_{iz}h_{jz}.\\
&s.t.\ H>0,\ and\ \sum_{p=1}^{M}u_p=1,u_p\geq 0\ for\ p=1,2,...,M.
\end{aligned}
\tag{S8}
$$

Here $\alpha \in \mathbb{R}$ is the regularization parameter.

2 FIGURES

REFERENCES

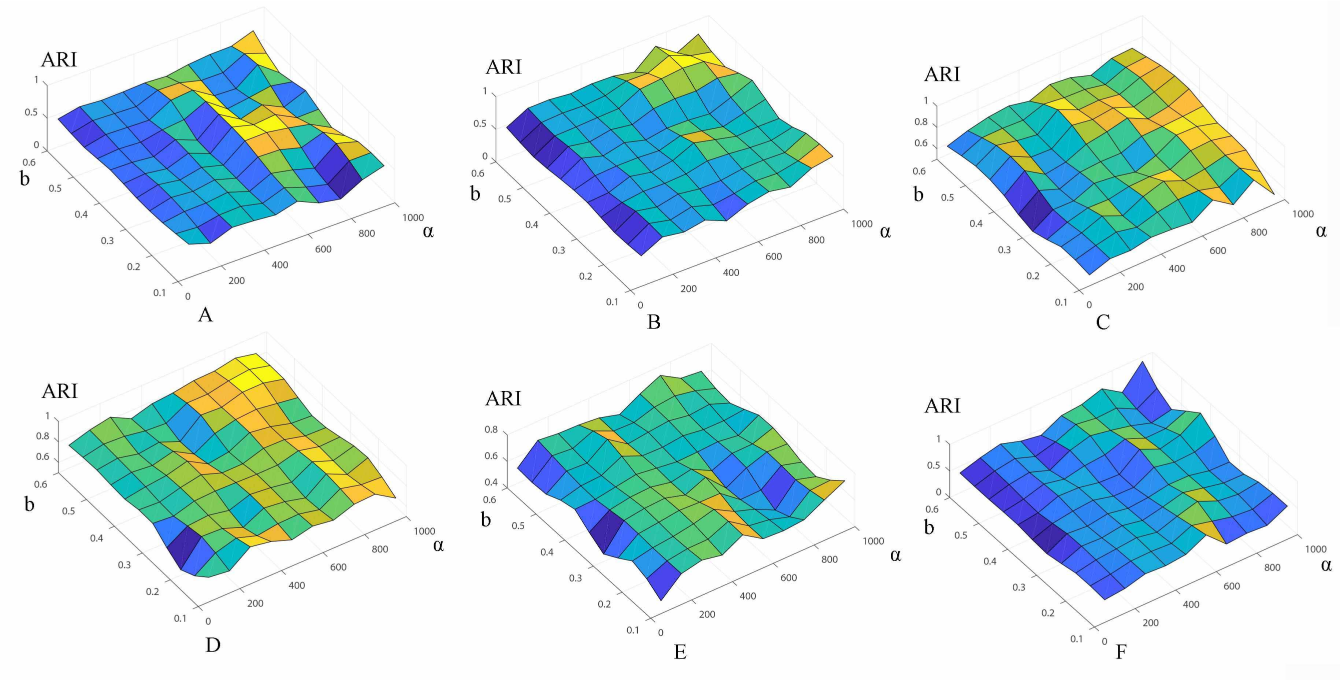

Figure S2. Performance of EC-PGMGR on 6 benchmark data sets with respect to different values of b and α measured in terms of the ARI score. The x-axis denotes the value of α, the y-axis denotes the value of b, and the z-axis denotes the value of the ARI. (A)Baron-human1. (B)Baron-human2. (C)Baron-human3. (D)Baron-human4. (E)Baron-mouse1. (F)Baron-mouse2.

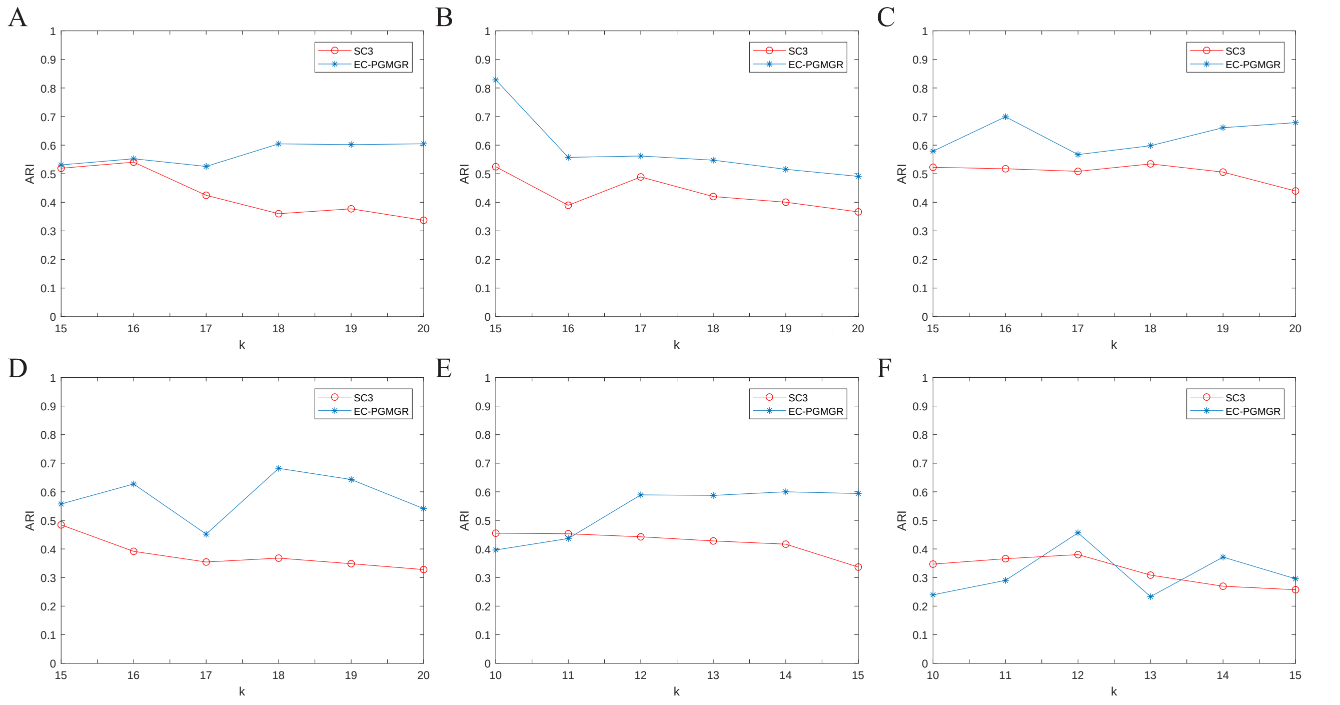

Figure S3. Assessing the robustness of EC-PGMGR for all data sets when varying the number of clusters for SC3 results and holding the other three individual methods constant. Different k will influence the results of SC3. (A) Baron-human1. (B) Baron-human2. (C) Baron-human3. (D) Baron-human4. (E) Baron-mouse1. (F) Baron-mouse2.

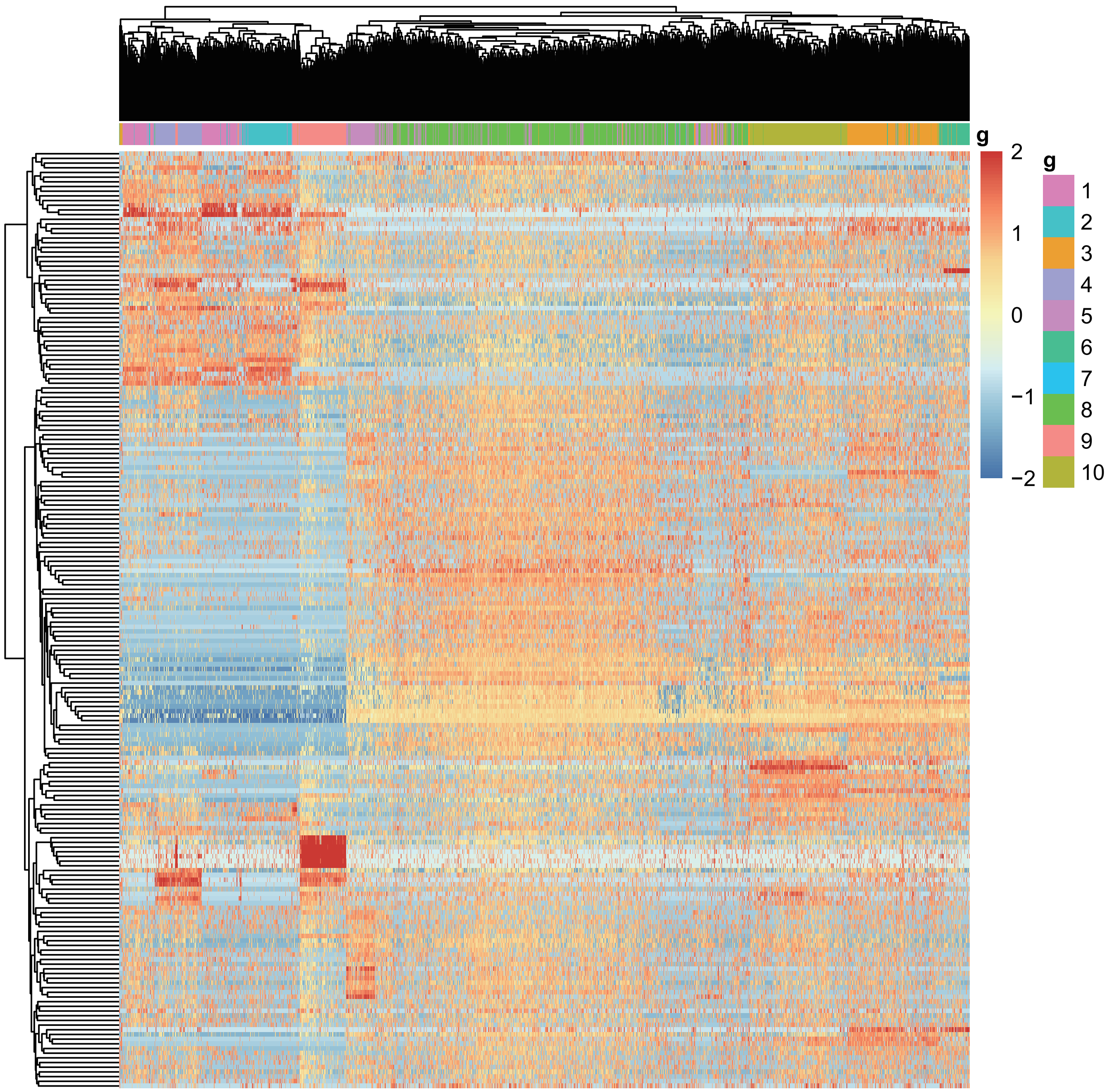

Figure S4. The heat map shows the top 200 standard deviation genes in the Baron-human1 experimental results. Each row represents the genes and each column represents the cells.

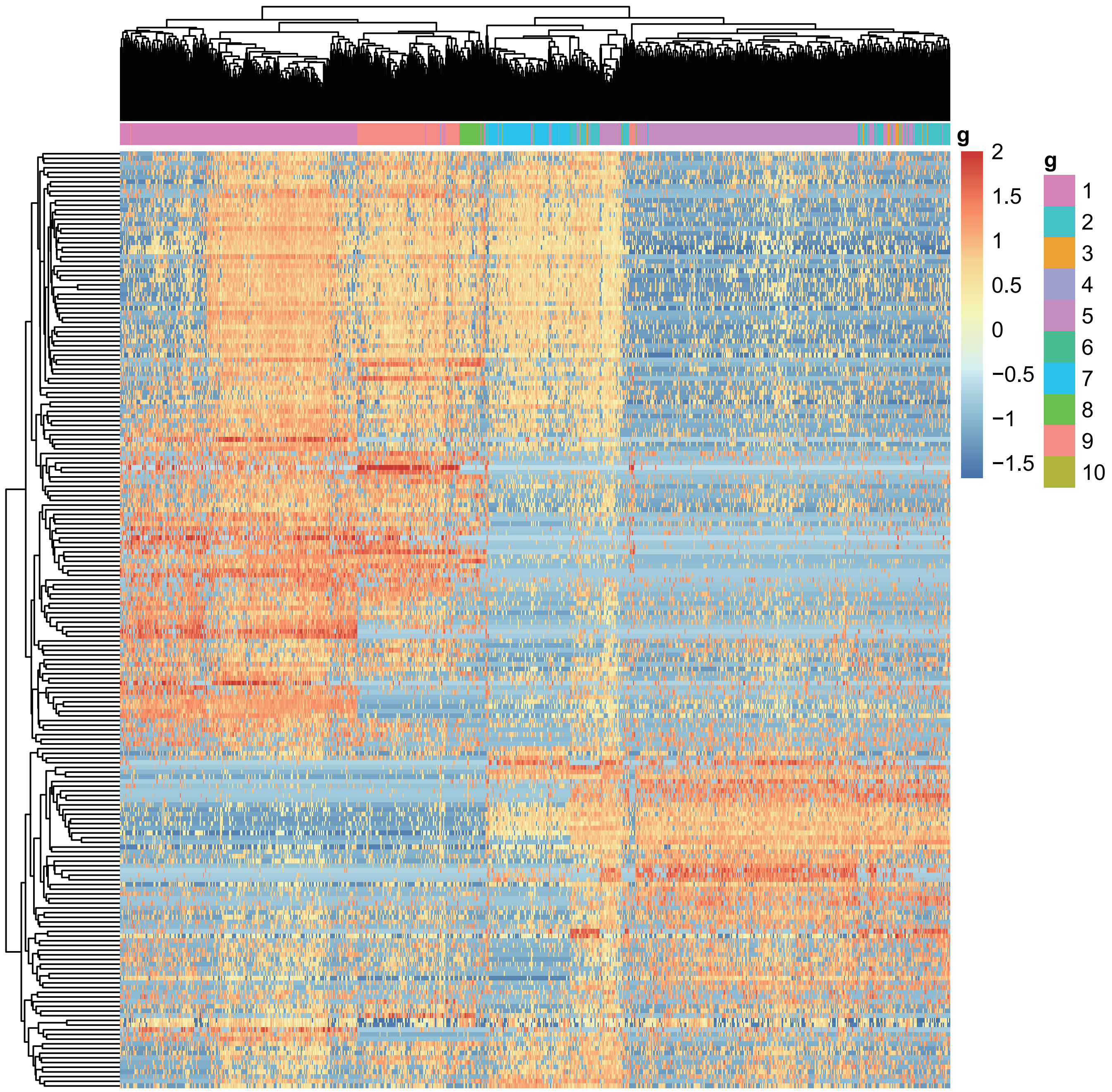

Figure S5. The heat map shows the top 200 standard deviation genes in the Baron-mouse1 experimental results. Each row represents the genes and each column represents the cells.
